# Somatostatin interneuron fate-mapping and structure in a Pten knockout model of epilepsy

**DOI:** 10.3389/fncel.2024.1474613

**Published:** 2024-10-21

**Authors:** Austin W. Drake, Lilian G. Jerow, Justin V. Ruksenas, Carlie McCoy, Steve C. Danzer

**Affiliations:** ^1^Department of Anesthesia, Cincinnati Children’s Hospital Medical Center, Cincinnati, OH, United States; ^2^Neuroscience Graduate Program, University of Cincinnati College of Medicine, Cincinnati, OH, United States; ^3^Medical Scientist Training Program, University of Cincinnati College of Medicine, Cincinnati, OH, United States; ^4^Neuroscience Research Center, Cincinnati Children’s Hospital Medical Center, Cincinnati, OH, United States

**Keywords:** somatostatin interneurons, epilepsy, mammalian target of rapamycin (mTOR), morphology, phosphatase and tensin homolog (Pten), mTORopathies, transgenic mice

## Abstract

Disruption of inhibitory interneurons is common in the epileptic brain and is hypothesized to play a pivotal role in epileptogenesis. Abrupt disruption and loss of interneurons is well-characterized in status epilepticus models of epilepsy, however, status epilepticus is a relatively rare cause of epilepsy in humans. How interneuron disruption evolves in other forms of epilepsy is less clear. Here, we explored how somatostatin (SST) interneuron disruption evolves in quadruple transgenic Gli1-CreER^T2^, Pten^fl/fl^, SST-FlpO, and frt-eGFP mice. In these animals, epilepsy develops following deletion of the mammalian target of rapamycin (mTOR) negative regulator phosphatase and tensin homolog (Pten) from a subset of dentate granule cells, while downstream Pten-expressing SST neurons are fate-mapped with green fluorescent protein (GFP). The model captures the genetic complexity of human mTORopathies, in which mutations can be restricted to excitatory neuron lineages, implying that interneuron involvement is later developing and secondary. In dentate granule cell (DGC)-Pten knockouts (KOs), the density of fate-mapped SST neurons was reduced in the hippocampus, but their molecular phenotype was unchanged, with similar percentages of GFP+ cells immunoreactive for SST and parvalbumin (PV). Surviving SST neurons in the dentate gyrus had larger somas, and the density of GFP+ processes in the dentate molecular layer was unchanged despite SST cell loss and expansion of the molecular layer, implying compensatory sprouting of surviving cells. The density of Znt3-immunolabeled puncta, a marker of granule cell presynaptic terminals, apposed to GFP+ processes in the hilus was increased, suggesting enhanced granule cell input to SST neurons. Finally, the percentage of GFP+ cells that were FosB positive was significantly increased, implying that surviving SST neurons are more active. Together, findings suggest that somatostatin-expressing interneurons exhibit a combination of pathological (cell loss) and adaptive (growth) responses to hyperexcitability and seizures driven by upstream Pten KO excitatory granule cells.

## Graphical Abstract

Illustration and summary of key study findings. The left portion of the illustration depicts DGC and SST interneuron interaction in normal conditions. The right portion depicts changes in the setting of DGC-Pten KO which are supported by the following findings in this study: (1) eGFP-SST cell density was reduced in multiple hippocampal regions. Remaining hilar eGFP-SST cells exhibited larger soma sizes. (2) Preserved eGFP-SST processes in the hilus and molecular layer, coupled with cell loss and an enlarged molecular layer, suggests sprouting. (3) Increased apposition of Znt-3 puncta to eGFP-SST processes suggests increased DGC innervation of SST interneurons. Created in BioRender.com. BioRender.com/h33m167. 
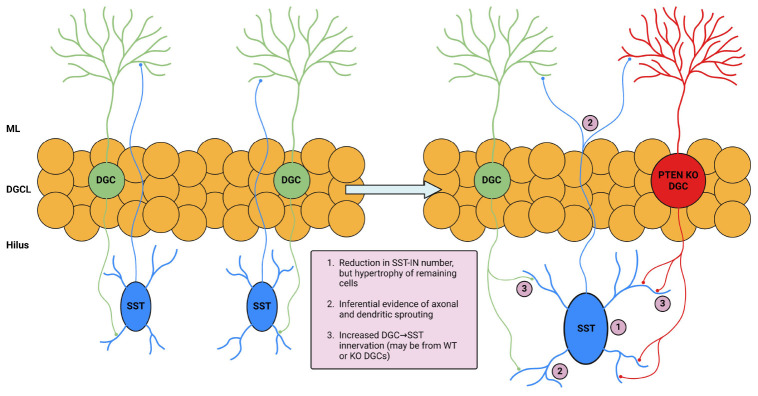

## Introduction

Hippocampal inhibitory interneurons have garnered significant interest for their potential role in epileptogenesis. Interneuron loss has long been identified as a hallmark pathology of epilepsy in both patients and animal models (Margerison and Corsellis, 1966; de Lanerolle et al., 1989; de Lanerolle et al., 2012; Buckmaster et al., 2017). Surviving interneurons, on the other hand, can undergo morphological and functional changes, possibly as a homeostatic response to maintain inhibitory control (Buckmaster and Dudek, 1997; Tóth et al., 2007; Halabisky et al., 2010). Much of the work describing interneuron loss and reorganization, however, has come from status epilepticus models, which may not be reflective of how interneurons are affected in epilepsies stemming from other etiologies.

Mutations in the mechanistic target of rapamycin (mTOR) pathway in the central nervous system produce a class of disorders, collectively called mTORopathies, which commonly cause childhood epilepsy (Crino, 2015). These diseases appear to develop in many patients following somatic mutations in excitatory neuron progenitors, such that interneurons are not directly impacted (Chung et al., 2023). Affected excitatory neurons undergo excessive sprouting, become hyperactive, and are postulated to drive seizures (Kwon et al., 2001; Kwon et al., 2006; Meikle et al., 2007). Despite apparent sparing of interneurons from the disease-causing mutations, however, extensive interneuron loss and restructuring are evident in patient samples (Spreafico et al., 1998; Thom et al., 2003; Nakagawa et al., 2017; Liang et al., 2020; Yan et al., 2023). Notably, mTORopathy models in which mutations are targeted to excitatory neuron lineages also show interneuron involvement (Zhong et al., 2021; Santos et al., 2017; LaSarge et al., 2021). How and why interneurons are affected in these settings remains to be elucidated and could provide critical information for understanding epileptogenic mechanisms.

Our group previously developed a mouse model of epilepsy utilizing a transgenic cre-lox approach in which epilepsy is induced by deletion of phosphatase and tensin homolog (Pten) from postnatally generated excitatory dentate granule cells (DGCs) (Pun et al., 2012). Loss of Pten, a negative regulator of the mTOR pathway, results in the development of cortical seizures when DGC-Pten knockout (KO) rates exceed 10%. Affected granule cells develop morphological and physiological abnormalities reminiscent of patient tissue samples (Franck et al., 1995; Parent et al., 2006; Williamson and Patrylo, 2007) and other models of epilepsy (Spigelman et al., 1998; Murphy et al., 2012). Using this model, we take advantage of the well-defined hippocampal circuitry to examine how KO cells affect downstream targets. Specifically, hippocampal somatostatin-expressing (SST) interneurons, known as “hilar perforant path (HIPP) cells,” are vulnerable in this model despite being spared from Pten deletion (LaSarge et al., 2021). Affected SST interneurons, located in the dentate hilus, are part of a negative feedback circuit, in which excitatory DGC input drives SST feedback inhibition to DGC distal dendrites (Freund and Buzsáki, 1996). Hilar SST neurons, therefore, are directly regulated by upstream Pten KO DGCs and are well-positioned to exert inhibitory control over these same aberrant, hyperexcitable DGCs.

To visualize SST interneurons in DGC-Pten KO mice, we developed a quadruple transgenic approach combining cre-mediated deletion of Pten from DGCs with flp-recombinase (FlpO)-mediated expression of enhanced green fluorescent protein (eGFP) in SST neurons. Using this fate-mapping approach, we assessed SST interneuron density, structure, molecular phenotype, and innervation patterns in control and DGC-Pten KO mice.

## Materials and methods

### Animals

Gli1-CreER^T2^, Pten^fl/fl^, SST-FlpO, and frt-eGFP mice, all on a C57BL/6 background, were acquired from The Jackson Laboratory and maintained within our colony (Table 1). Animal lines were crossed to produce quadruple transgenic mice, along with littermate controls (Table 2). The combination of Gli1-CreER^T2^ and Pten^fl/fl^ allowed for tamoxifen-dependent deletion of Pten from postnatally generated dentate granule cells (Singh et al., 2015; LaSarge et al., 2016). Tamoxifen was given subcutaneously (dose: 250 mg/kg, preparation: dissolved in corn oil at a concentration of 20 mg/ml) to all mice in the study on postnatal day 14. This dosing regimen results in Pten deletion from >10% of DGCs (LaSarge et al., 2019). The addition of SST-FlpO, which drives flp-recombinase (FlpO) expression under control of the SST promoter, and frt-eGFP, a FlpO-dependent reporter, to these mice enabled endogenous expression of enhanced green fluorescent protein (eGFP) in somatostatin-expressing cells (eGFP-SST). Study animals from both groups were housed together (up to 4 mice per cage) in our colony room under a 14:10 h light/dark cycle. All studies were designed to minimize animal distress and were performed in alignment with CCHMC Institutional Animal Care and Use Committee (IACUC) and National Institutes of Health (NIH) guidelines.

**TABLE 1:** Identification of transgenic mouse lines and key study resources.

Animal lines and key resources	RRID
Gli1-CreER^T 2^ Transgenic Mouse	IMSR_JAX:007913
Pten Transgenic Mouse	IMSR_JAX:006440
SST-FlpO Transgenic Mouse	IMSR_JAX:028579
eGFP Transgenic Mouse	MMRRC_032038-JAX
Allen Mouse Brain Reference Atlas	SCR_002978
Nikon A1R Confocal Laser Scanning Microscope	SCR_020317
NIS-Elements AR (Version 5.42.03)	SCR_014329
Cincinnati Children’s Bio-Imaging and Analysis Facility	SCR_022628
Imaris (Version 10.1.1)	SCR_007370
Neurolucida 360	SCR_016788
Neurolucida Explorer	SCR_017348
GraphPad Prism (Version 10)	SCR_002798

**TABLE 2:** Study group details.

Group	Genotype	Number
DGC-Pten knockout	Gli1-CreER^T 2+/–^, Pten^fl /fl^, SST-FlpO^+/–^, and frt-eGFP^+/–^	6 (3M, 3F)
Control	Gli1-CreER^T 2–/–^, Pten^fl /fl^, SST-FlpO^+/–^, and frt-eGFP^+/–^	5 (3M, 2F)

### Tissue collection and processing

At 10 weeks of age, mice were deeply anesthetized with pentobarbital (100 mg/kg, i.p.). Upon full sedation, mice underwent transcardial perfusion for 1 min with ice-cold phosphate-buffered saline (PBS, pH 7.4) + 0.1% heparin, followed by 10 min of paraformaldehyde (PFA) solution (2.5% PFA, 4% sucrose, 0.1M PBS, pH 7.4). Following perfusion, brains were collected and refrigerated at 4°C in PFA solution overnight. The brains were subsequently cryoprotected through storage in sucrose solutions of increasing concentration (10%, 20%, and 30%). Following cryoprotection, brains were frozen in 2-methylbutane cooled to −25°C, bisected, and stored at −80°C. Brains were coronally cryosectioned at a 40 μm thickness onto gelatin-coated slides in a 1 in 12 stereological series. Slides were stored at −80°C.

### Immunohistochemistry and imaging

Sections were immunostained for various targets and all antibodies were evaluated for cross-reactivity prior to use in this study (Table 3). All immunohistochemistry experiments utilized the following 3-day protocol: slides were permeabilized overnight in 3% Triton-100, 0.75% glycine in 0.1M PBS at room temperature. The following day they were washed in 0.1M PBS, placed in blocking solution (5% normal goat or donkey serum, 0.75% glycine, and 1.5% Triton-100 in 0.1M PBS) for at least 1 h, then immersed in blocking solution containing the primary antibodies of interest overnight at room temperature. On the final day, slides were incubated in blocking solution containing secondary antibodies for 4 h at room temperature, washed with 0.1M PBS and coverslipped with ProLong Glass Antifade Mountant with NucBlue (Thermo Fisher Scientific, Catalog Number: P36981). For each immunostaining protocol, one slide from each animal, mounted with 2–4 brain hemisections collected in a 1 in 12 series (480 μm between sections) through the dorsal-ventral extent of the hippocampus (bregma −1.30 to −3.30 mm), was stained. For each antibody, slides with matching bregma coordinates were selected. Confocal images were collected from all sections on a slide for analysis. All confocal imaging in this study was conducted using the same Nikon A1R inverted microscope.

**TABLE 3:** Primary and secondary antibodies.

Antibody	Target	Species	Concentration	RRID
Primaries	Somatostatin	Rabbit	1:200	AB_2789834
	c-Fos	Rabbit	1:1,000	AB_2106783
	FosB	Rabbit	1:1,000	AB_2721123
	Pten	Rabbit	1:250	AB_390810
	Znt3	Rabbit	1:500	AB_2189664
	Nitrotyrosine	Rabbit	1:500	AB_310089
	Cleaved caspase-3	Rabbit	1:400	AB_2341188
	Parvalbumin	Guinea pig	1:1,000	AB_2156476
	GFP	Chicken	1:500	AB_300798
Secondaries	Alexa Fluor 647 anti-rabbit IgG (H+L)	Donkey	1:750	AB_2536183
	Alexa Fluor 568 anti-chicken IgG (H+L)	Donkey	1:750	AB_2921072
	Alexa Fluor 647 AffiniPure Donkey Anti-Guinea Pig IgG (H+L)	Donkey	1:750	AB_2340476
	Alexa Fluor 647 anti-rabbit IgG (H+L)	Goat	1:750	AB_2535813
	Alexa Fluor 568 anti-chicken IgG (H+L)	Goat	1:750	AB_2534098

### Determination of DGC-Pten KO rates

Immunostaining for Pten was done to determine the percentage of Pten KO DGCs. Images were taken at the midpoints of the dentate gyrus upper and lower blades using a 60× water objective (NA 1.27, steps: 10, step size: 0.5 μm, resolution: 0.21 μm/pixel). The total number of NucBlue-stained cells within the granule cell layer were counted, excluding those at the image boundaries. The number of NucBlue+ cells that were Pten-negative were also counted, allowing for the percentage of Pten KO DGCs to be calculated as follows: (total number of NucBlue+, Pten-negative cells divided by the total number of NucBlue+ cells).

### SST fate-mapping

To determine the sensitivity and specificity of eGFP-labeling of SST interneurons, sections were immunostained for SST. Confocal images of the entire dentate hilus were collected from 2 to 4 sections per mouse using a 20× water objective (NA 0.95, steps: 10, step size: 1 μm, resolution: 1.24 μm/pixel). Images were imported into Imaris software (Version 10.1.1) to determine colocalization between eGFP and SST. In Imaris, the spots feature with background subtraction was used to identify both eGFP+ and SST+ cells, as well as identify colocalized cells. Overlap between eGFP+ and PV+ cells was also examined using this approach in Imaris.

### SST cell density measurements

To determine the density of eGFP-SST interneurons in hippocampal subregions, a confocal tile-scan was run to capture the entire hippocampus in 2–4 sections per mouse using a 20× water objective (NA 0.95, steps: 10, step size: 2 μm, resolution: 1.24 μm/pixel). Images were imported into Imaris software for analysis. Regions of interest (ROIs) encompassing the entire hippocampus, hilus, CA1, and CA3 were manually traced as surfaces using the bregma-matched images from the Allen Mouse Brain Atlas (2004) to define regions. The hilus was defined by the hilar/granule cell body layer border and a line drawn between the tips of the upper and lower blades of the dentate granule cell layer, excluding the pyramidal layer. CA3 was defined by the same line between the tips of the dentate blades, the alveus, and a line drawn from the tip of the upper blade to the alveus based upon the bregma-matched boundary in the Atlas. The borders of CA1 were defined by the hippocampal fissure, alveus, and a line drawn perpendicular to the pyramidal layer. Spot detection was used to identify eGFP+ cells in each ROI. Area measurements were used to calculate the volume of each ROI for determination of eGFP+ cell density.

### 3-Nitrotyrosine and cleaved caspase-3 analyses

The entire dentate hilus was imaged using a 20× water objective (NA 0.95, steps: 10, step size: 1 μm, resolution: 1.24 μm/pixel) on tissue immunostained for either 3-nitrotyrosine (3-NT) or cleaved caspase-3. Cell counts were performed on 2–4 hilar regions per mouse in Imaris using spot detection to quantify the percentage of eGFP+ cells that were also 3-NT+ or cleaved caspase-3+. Cells were considered 3-NT+ or cleaved caspase-3+ if somatic fluorescence intensity was at least 2 times greater than background.

### Morphology measurements

To determine the soma area and proximal dendrite number of eGFP-SST interneurons in the hilus, images of the whole hilus through the full depth of each 40 μm section were obtained using a 60× water objective (NA 1.27, step size: 0.5 μm, resolution: 0.41 μm/pixel). Confocal image stacks were then imported into Neurolucida 360 software for reconstruction (MicroBrightField Inc.). Each hilar eGFP+ cell was screened to evaluate whether it met inclusion criteria for reconstruction. Only cells located within the hilus (>10 μm) from the hilar-granule cell layer border were reconstructed, so as to exclude basket cells located in the subgranular zone/granule cell body layer. In addition, only cells for which the midpoint of the soma was contained within the z-stack were included, thus excluding partial soma profiles. All cells that met criteria were reconstructed, with a minimum of 15 cells per mouse. Neurolucida 360 was used to encode maximal soma profile area, while the number of proximal dendrites was manually counted for each cell.

The density of eGFP-SST neurites was assessed in both the molecular layer and the hilus in 2–4 sections per mouse. For the molecular layer, images were collected from the midpoint of the upper blade capturing inner, middle and outer lamina of the molecular layer (NA 1.27, step size: 0.5 μm, steps: 10, resolution: 0.2 μm/pixel). Confocal image stacks were analyzed in NIS-Elements AR (Version 5.42.03). For analyses, ROIs containing the inner, middle, and outer molecular layer lamina were defined using proportions of 17%, 41.5%, and 41.5% of the molecular layer area, respectively (Santos et al., 2011). The percentage of the area of each region occupied by eGFP+ processes was then computed using the Object Count tool in NIS-Elements AR using identical settings for each measurement. Hilar images were analyzed in an analogous manner to assess eGFP-SST interneuron process coverage within the hilus. Area occupied by eGFP-SST interneuron somas was excluded from the hilar ROI.

### Apposition between Znt3 puncta and GFP+ processes

For immunohistochemical assessment of granule cell innervation onto eGFP-SST interneurons, 2–4 sections per mouse were immunostained for the granule cell presynaptic terminal marker zinc transporter 3 (Znt3) (McAuliffe et al., 2011). Images of the center of the hilus were taken using a 100× oil objective (NA 1.45, steps: 4, step size: 0.125 μm, resolution: 0.25 μm/pixel, dimensions: 128 μm × 128 μm) and imported into Imaris software. Imaris was used to measure soma profile circumference of eGFP-SST cells and dendrites were traced using the filaments feature. Znt3 puncta were identified with the spot detection feature using an estimated diameter of 0.5 μm. All puncta located within 0.2 μm of an eGFP+ process or cell body were scored as being in contact. Total eGFP-SST dendrite length was added to total eGFP-SST cell circumference for each hilar ROI to compute an overall eGFP-SST interneuron length available for contact with Znt3 puncta.

### c-Fos and FosB labeling

To assess surrogate markers of neuronal activity for DGCs and eGFP-SST interneurons, immunostaining was conducted for c-Fos and FosB. Images of the entire hilus from 2 to 4 sections/mouse taken on a 20× water objective (NA 0.95, steps: 10, step size: 1 μm, resolution: 1.24 μm/pixel) were used to examine c-Fos and FosB in eGFP-SST interneurons and c-Fos in DGCs. To accommodate the higher density of labeling, images of the midpoint of the upper and lower blades of the granule cell layer were collected to look at FosB in DGCs using a 60× water objective (NA 1.27, steps: 10, step size: 1 μm, resolution: 0.41 μm/pixel). These images were imported into Imaris software, where spot detection was used to identify c-Fos+ or FosB+ eGFP-SST interneurons and hippocampal granule cells.

### Statistical analysis

Investigators were blind to animal genotype for all data collection and analysis. Although this study was not powered to establish sex differences, both male and female mice were used and binned for analyses. All data are reported as mean ± SEM, unless otherwise specified, and group differences with *p* < 0.05 were considered significant. Unless specified otherwise, Student’s *t*-tests were used for group comparisons. GraphPad Prism (Version 10) was used for all statistical analyses.

### Figure preparation

NIS-Elements AR (Version 5.42.03) was used to process representative images via cropping and contrast adjustments. All compared images were subject to identical modification. Plots were generated using GraphPad Prism (Version 10). BioRender was used to create the Graphical Abstract.

## Results

### eGFP fate-mapping of SST interneurons in DGC-Pten KO mice

To visualize SST interneurons in mice with mosaic loss of Pten from a subset of hippocampal dentate granule cells (DGC-Pten KOs), we developed a quadruple-transgenic approach combining cre-lox technology to delete Pten from granule cells (Gli1-CreER^T2+/–^, Pten^fl/fl^) with flp-frt technology to express eGFP in SST interneurons (SST-FlpO, frt-eGFP). Quadruple transgenic Gli1-CreER^T2+/–^, Pten^fl/fl^, SST-FlpO^+/–^, frt-eGFP^+/–^ mice were treated with tamoxifen on P14 to activate cre recombinase and delete Pten from >10% of granule cells. Gli1-CreER^T2^ negative (Gli1-CreER^T2–/–^, Pten^fl/fl^, SST-FlpO^+/–^, frt-eGFP^+/–^) littermates with intact Pten expression, also treated with tamoxifen in P14, were used as controls for all experiments.

**FIGURE 1:**
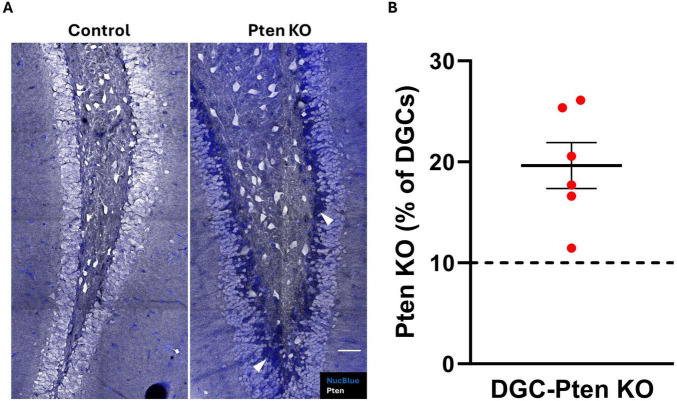
Pten knockout from dentate granule cells. **(A)** Confocal images showing the dentate granule cell layer and hilus in control and DGC-Pten KO mice. Knockout cells are identified by lack of Pten immunostaining, evident as a dark blue band of Nuclear Blue counterstained neurons concentrated predominantly along the hilar-granule cell layer border. Examples of KO cells are indicated by the arrowheads (white). Scale, 50 μm. **(B)** Percentage of KO dentate granule cells for each DGC-Pten KO mouse. Bars represent animal means ± SEM.

To confirm Pten deletion in DGC-Pten KO mice, brain sections were immunostained for Pten. Consistent with prior studies, a clear band of Pten immunonegative granule cells was evident in DGC-Pten KOs (Figure 1A), reflecting deletion among postnatally generated granule cells which occupy the inner 1/3 of the granule cell body layer. Cell counts to determine the percentage of the granule cell population that lacked Pten revealed that deletions exceeded 10% for all DGC-Pten KO animals (Figure 1B; mean 19.64% ± 2.27%). Previously, work in this model has established that this exceeds the KO cell load for the development of spontaneous seizures. These seizures typically begin around 6–8 weeks of age and occur at a rate of approximately 1–2 seizures per day (Pun et al., 2012; LaSarge et al., 2021).

**FIGURE 2:**
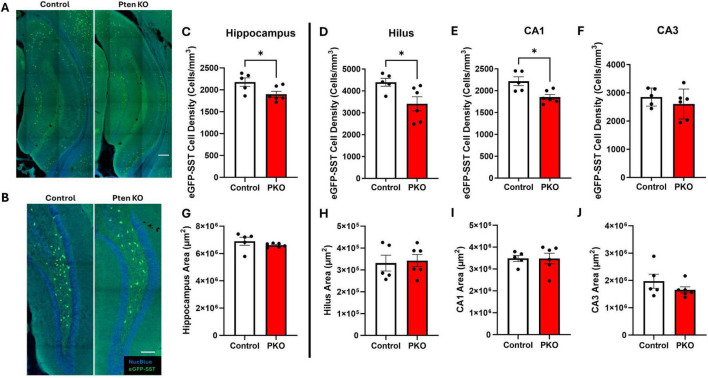
Reduced SST interneuron density in the hippocampus. **(A)** Confocal images of hippocampus showing the distribution of eGFP+ cells in Gli1-CreER^T 2–/–^, Pten^fl /fl^, SST-FlpO^+/–^, frt-eGFP^+/–^ (control) and Gli1-CreER^T 2+/–^, Pten^fl /fl^, SST-FlpO^+/–^, frt-eGFP^+/–^ (DGC-Pten KO) mice. Scale, 200 μm. **(B)** eGFP+ cells within the hilus illustrating reduced cellular density in DGC-Pten KO mice. Scale, 100 μm. **(C–F)** Density of eGFP-SST interneurons in the hippocampus, hilus, CA1, and CA3, respectively. **(G–J)** Areas of the hippocampus, hilus, CA1, and CA3, respectively. *p < 0.05. Bars represent animal means ± SEM.

Enhanced green fluorescent protein labeling of presumptive SST interneurons (termed eGFP-SST) was robust in both control and DGC-Pten KO mice. The density, distribution, and morphological features of eGFP-positive cells were consistent with prior work describing SST interneurons (Freund and Buzsáki, 1996; Hosp et al., 2014), although within the dentate gyrus, infrequent granule cells labeled with GFP were observed (Figures 2A, B). Prominent SST fiber pathways in the CA1 stratum lacunosum-moleculare and the dentate middle and outer molecular layers were also evident (Figure 2A). eGFP-labeled cells appeared healthy in control and DGC-Pten KO mice, without evidence of cellular injury (e.g., pyknotic cell bodies or beaded dendrites).

### The density of eGFP+ SST interneurons is reduced in DGC-Pten KOs

Prior stereological estimates revealed reduced numbers of SST immunoreactive neurons in DGC-Pten KO mice (LaSarge et al., 2021). Since reductions revealed by immunohistochemistry could reflect either interneuron loss or reduced SST expression, we queried whether eGFP fate-mapping would produce a similar result. For this analysis, we measured the density of eGFP-SST interneurons for the entire hippocampus and hippocampal subregions – dentate hilus, CA1, and CA3 – containing large populations of SST neurons. Consistent with prior findings, we observed a reduction in hippocampal eGFP-SST interneuron density in DGC-Pten KO mice (Figure 2C; Control, 2,176 ± 97 cells/mm^3^; KO, 1,901 ± 68 cell/mm^3^; *p* = 0.0402). Additionally, there was a significant reduction in eGFP-SST cell density in the hilus (Figure 2D; Control, 4,395 ± 182 cell/mm^3^; KO, 3,411 ± 322 cells/mm^3^; *p* = 0.0334) and CA1 (Figure 2E; Control, 2,217 ± 101 cell/mm^3^; KO, 1,853 ± 59 cells/mm^3^; *p* = 0.0102), but not in CA3 (Figure 2F; Control, 2,852 ± 143 cells/mm^3^; KO, 2604 ± 217 cells/mm^3^; *p* = 0.3860). Since reductions in density could reflect reduced neuron numbers or increased tissue volume, we also assessed whether our ROIs exhibited any expansion in DGC-Pten KOs. There were no statistically significant volume differences between control and DGC-Pten KO groups for the entire hippocampus or the sub-regions used for cell density comparisons (Figures 2G–J). Findings indicate that SST interneuron loss extends beyond hilar interneurons directly innervated by Pten KO cells to include interneurons in CA1 as well.

### 3-Nitrotyrosine and cleaved caspase-3 labeling is unchanged among SST interneurons

**FIGURE 3:**
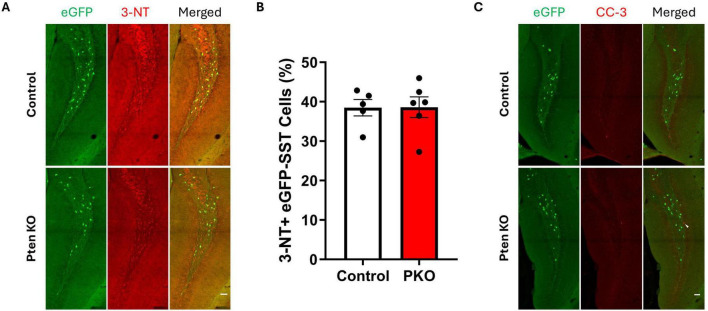
Expression of 3-nitrotyrosine and cleaved caspase-3 is similar between control and DGC-Pten KO mice. **(A)** Representative images of 3-NT immunostaining of hilar eGFP-SST interneurons in control and DGC-Pten KO groups. Scale, 50 μm. **(B)** The percentage of 3-NT+ hilar eGFP-SST interneurons did not differ significantly between groups. **(C)** Representative images of cleaved caspase-3 (CC-3) immunostaining of the dentate gyrus in control and DGC-Pten KO groups. The arrowhead denotes a CC-3+, GFP- cell in the dentate gyrus granule cell layer. Scale, 50 μm. Bars represent animal means ± SEM.

Reduced eGFP-SST interneuron density in DGC-Pten KOs implies these neurons may be dying, perhaps as a secondary effect of seizures. We queried, therefore, whether surviving eGFP-SST interneurons would show signs of cellular stress. To examine this possibility, sections were immunostained with 3-nitrotyrosine (Figure 3A), which is generated through nitration of tyrosine residues and can be indicative of cell damage (Cobb and Cole, 2015). Staining for cleaved caspase-3, a marker for apoptosis, was also performed to assess cell death. However, there was no difference in the proportion of 3-NT+, eGFP-SST cells in the dentate hilus between DGC-Pten KOs and controls (Figure 3B; Control, 38.47% ± 2.08% of eGFP+ cells were 3-NT+; KO, 38.61% ± 2.62%; *p* = 0.9684), and no colocalization of eGFP and cleaved caspase-3 was observed (Figure 3C; Control, 0.0% ± 0.0% of eGFP+ cells were cleaved caspase-3+; KO, 0.0% ± 0.0%).

### The molecular phenotype of SST neurons is unchanged in DGC-Pten KO mice

**FIGURE 4:**
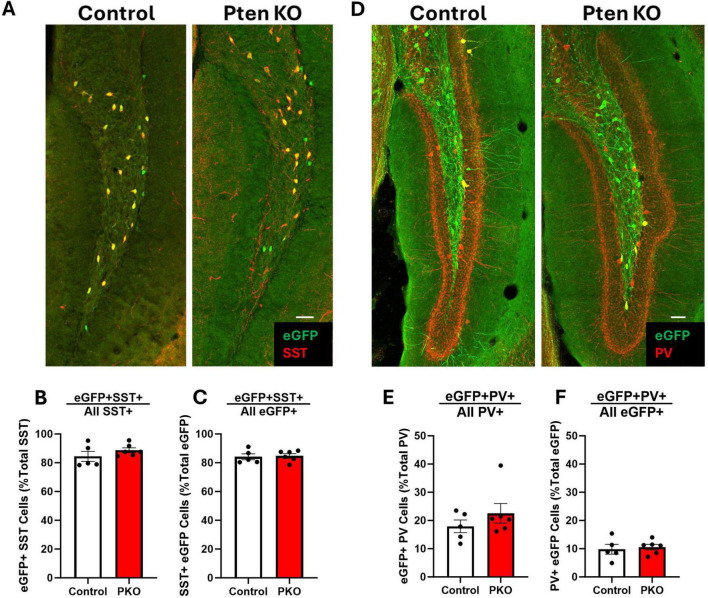
Preserved molecular phenotype of SST neurons in DGC-Pten KO mice. **(A)** Representative images of the hilus showing overlap between eGFP-labeled cells and SST immunostaining. Scale, 50 μm. **(B)** The percentage of hilar SST-immunoreactive cells that co-express eGFP. **(C)** Graph shows the percentage of hilar eGFP+ cells that were immunopositive for SST. **(D)** Representative images of the hilus showing overlap between eGFP-labeled cells and PV immunostaining. Scale, 50 μm. **(E)** The percentage of hilar PV+ cells that co-express eGFP. **(F)** The percentage of hilar eGFP+ cells that also express PV. Bars represent animal means ± SEM.

Somatostatin interneurons are routinely identified by immunostaining for SST. Since SST expression is activity dependent, however, and populations might shift through selective loss of cells with either more or less SST, reliance on SST immunostaining to characterize SST neurons can produce misleading results. The transgenic approach employed in this study enables permanent eGFP labeling of cells expressing SST-driven flp-recombinase regardless of whether the cells continue to express SST. This fate-mapping strategy, therefore, provides an opportunity to determine whether the proportion of interneurons expressing interneuron markers shifts from the time of initial SST expression through the development of seizures. The majority of SST-immunoreactive neurons in the hilus of both control and DGC-Pten KO mice were eGFP+, with no difference between groups (Figures 4A, B; Control, 84.47% ± 3.49%; KO, 88.72% ± 1.69%; *p* = 0.2758). Similarly, the majority of eGFP+ neurons were immunoreactive for SST, again with no difference between groups (Figure 4C; Control, 84.17% ± 2.07%; KO, 84.76% ± 1.54%; *p* = 0.8212). Findings are consistent with prior work demonstrating sensitivity and specificity of the approach (Kraus et al., 2022), and indicate that Pten deletion from granule cells, with resulting seizures, does not alter the proportion of interneurons expressing SST. Next, we assessed whether Pten deletion in DGCs altered the proportion of eGFP+ interneurons co-expressing the calcium binding protein and interneuron marker parvalbumin (PV). A minority of PV-immunoreactive neurons in the hilus were co-labeled with eGFP, with no difference between groups (Figures 4D, E; Control, 17.93% ± 2.25%; KO, 22.54% ± 3.49%; *p* = 0.3183). Similarly, a subset of eGFP+ neurons were immunoreactive for PV. This proportion was also not altered between control and DGC-Pten KO mice (Figure 4F; Control, 9.81% ± 1.73%; KO, 10.58% ± 0.99%; *p* = 0.6979).

### Hilar somatostatin-expressing interneurons exhibit increased soma area in DGC-Pten KO mice

**FIGURE 5:**
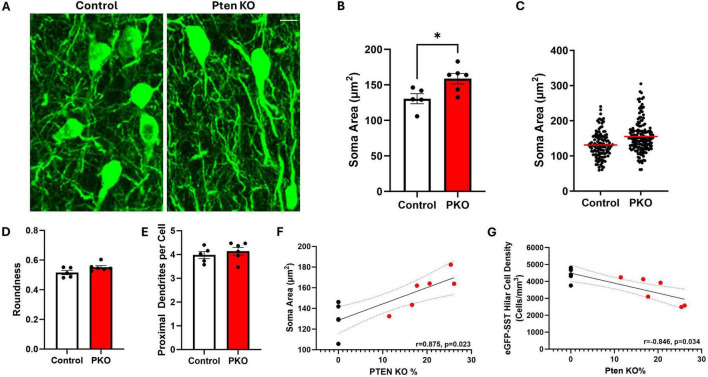
Hilar SST interneurons undergo morphological changes in DGC-Pten KO mice. **(A)** Representative images showing hilar eGFP-SST interneuron (HIPP) cell bodies and dendrites in control and DGC-Pten KO mice. Scale, 10 μm. **(B)** Graph illustrating the significant increase in eGFP-SST soma area in DGC-Pten KO mice. **(C)** Scatterplot showing the soma area for each reconstructed eGFP-SST interneuron in control (118 cells) and DGC-Pten KO (153 cells) mice. The red lines depict the mean soma areas. **(D)** Average soma roundness did not differ significantly between groups. **(E)** The number of primary dendrites per eGFP-SST interneuron did not differ significantly between groups. **(F)** Average soma area was positively correlated with the percentage of Pten KO DGCs in each mouse (*n* = 6 DGC-Pten KO mice). **(G)** Average hilar SST cell density was negatively correlated with the percentage of Pten KO DGCs in each mouse (*n* = 6 DGC-Pten KO mice). *p < 0.05. Bars represent animal means ± SEM.

Granule cells directly innervate SST neurons, which in turn provide feedback inhibition to granule cells (Milner and Bacon, 1989; Leranth et al., 1990). Loss of SST neurons could contribute to impaired inhibition in DGC-Pten KO mice. To begin to assess how this feedback inhibitory circuit is changing, the structure of eGFP-SST neurons was examined (Figure 5A). In DGC-Pten KO mice, there was a significant increase in the mean area of eGFP-SST somas (Figure 5B; Control, 130.46 ± 7.05 μm^2^; KO, 158.82 ± 7.36 μm^2^; *p* = 0.0225). Plotting all measured somas (rather than animal means) revealed that the entire population shifted to larger sizes (Figure 5C). There was no evidence for a subpopulation of eGFP-SST cells with unusually small somas, which could reflect dying, pyknotic cells. Hilar SST neurons typically have a fusiform shape to their somas. This pattern was not altered, as eGFP-SST soma roundness was similar between groups (Figure 5D; Control, 0.52 ± 0.01; KO, 0.55 ± 0.01; *p* = 0.0827). The mean number of proximal dendrites per eGFP-SST neuron was also similar between groups (Figure 5E; Control, 3.98 ± 0.15; KO, 4.14 ± 0.16; *p* = 0.4851). Interestingly, soma area was positively correlated with the percentage of DGC-Pten KO cells in each animal, while hilar cell density was negatively correlated [Figures 5F, G; Pearson correlations using only DGC-Pten KO mice, (soma area, *r* = 0.875, *p* = 0.023); (cell density, *r* = −0.846, *p* = 0.034)]. This suggests that higher DGC-Pten KO leads to greater SST interneuron loss, which in turn promotes a greater degree of compensatory sprouting and growth among surviving SST interneurons.

**FIGURE 6:**
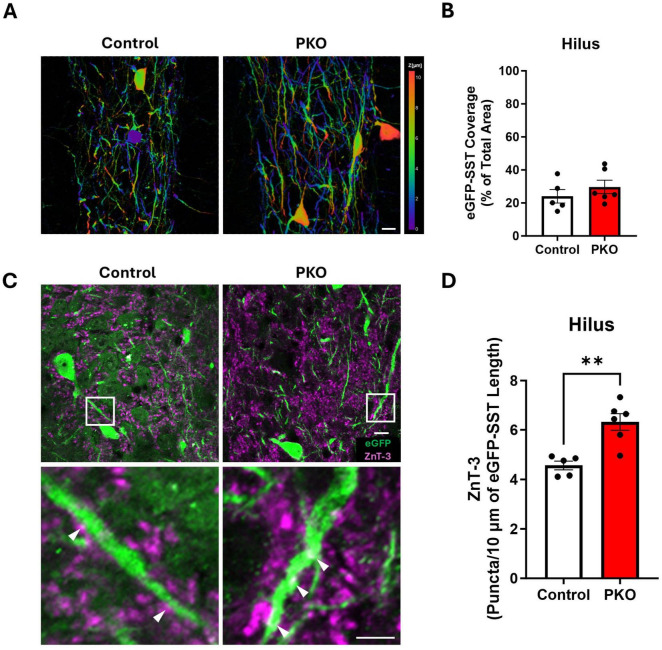
Increased apposition between Znt3 puncta and eGFP-SST interneuron processes. **(A)** Depth coded images of the hilus showing the area coverage of eGFP-SST processes. Scale, 20 μm. **(B)** Bar graph showing similar area coverage of eGFP-SST processes. **(C)** Representative images showing Znt3 puncta (purple) and eGFP-SST neurons in the dentate hilus. The lower panel shows magnified images from the regions in the upper panel indicated by white boxes. Arrowheads (white) indicate examples of apposition between Znt3 puncta and eGFP-SST processes. Scale, 10 μm. **(D)** Bar graph shows the significant increase in apposition of Znt3 puncta to eGFP-SST interneuron processes and cell bodies in DGC-Pten KO mice. **p < 0.01. Bars represent animal means ± SEM.

Evidence of somatic hypertrophy suggests that surviving eGFP-SST interneurons might undergo compensatory growth. The dendrites of hilar SST neurons are largely contained within the hilus and are innervated by granule cell mossy fiber axon collaterals (Leranth et al., 1990). Although it was not possible to trace the dendrites of individual eGFP-labeled SST neurons, we were able to quantify the percentage of the hilus occupied by eGFP-labeled processes, providing an indirect measure of the dendritic extent fielded by SST neurons (Figure 6A). Despite the reduction in the density of eGFP-SST somas, the percentage of the hilus occupied by eGFP+ processes did not significantly differ between groups (Figure 6B; Control, 24.07% ± 4.09%; KO, 29.74% ± 4.06%; *p* = 0.3551). Findings are consistent with the interpretation that surviving SST neurons have larger dendritic trees.

### Increased apposition between dentate granule cell presynaptic terminals and eGFP+ SST interneuron processes

Larger dendritic trees would provide increased opportunity for innervation by granule cells. To determine whether this might be the case, we immunolabeled granule cell presynaptic terminals with Zinc Transporter 3 (Znt3) (McAuliffe et al., 2011) and assessed apposition to eGFP-SST interneuron dendrites and cell bodies. The density of Znt3 puncta apposed to eGFP-labeled dendrites and somas in the hilus was significantly increased in DGC-Pten KO mice relative to controls (Figures 6C, D; Control, 4.56 ± 0.18 puncta/10 μm; KO, 6.32 ± 0.34 puncta, 10 μm; *p* = 0.0019), suggesting that granule cell input to SST neurons is increased.

### GFP+ fiber density is preserved in the dentate molecular layer of DGC-Pten KO mice

**FIGURE 7:**
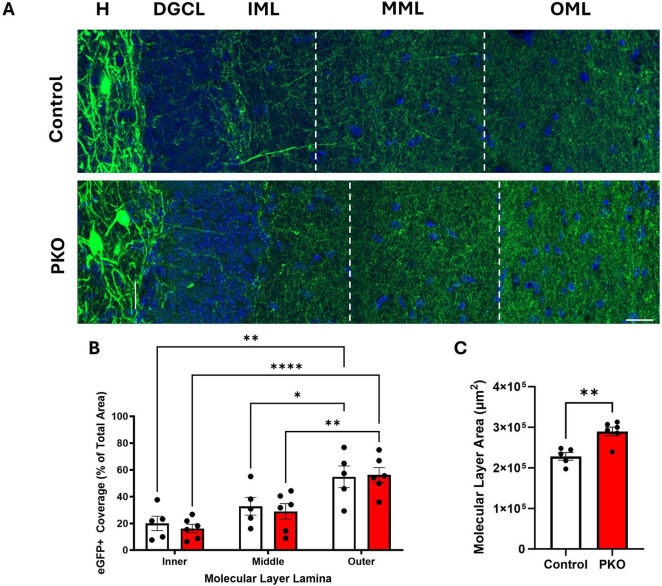
eGFP-SST interneuron processes density is maintained in DGC-Pten KO mice. **(A)** Representative images of the hilus (H), dentate granule cell body layer (DGCL), and dentate inner (IML), middle (MML), and outer molecular layers (OML) in control and DGC-Pten KO (PKO) mice. Scale, 20 μm. **(B)** Bar graph showing the area occupied by eGFP-SST interneuron processes in the IML, MML, and OML, respectively. **(C)** Bar graph depicting the significant increase in molecular layer area among DGC-Pten KO mice relative to controls. *p < 0.05, **p < 0.01, ****p < 0.0001. Bars represent animal means ± SEM.

In light of evidence that granule cells increase their input to eGFP-SST interneurons, we next queried whether eGFP-SST output to granule cells was altered. SST neurons provide feedback inhibition to the distal portions of granule cell dendritic trees, located in the dentate middle and outer molecular layers. With a similar approach to that used in the hilus, the percentage of molecular layer area (inner, middle, and outer lamina) occupied by eGFP-SST axons was quantified (Figures 7A, B). There was no significant effect of genotype but there was an effect of molecular layer lamina [two-way ANOVA, genotype main effect (*p* = 0.6636), lamina main effect (*p* < 0.0001)], and no correlations between eGFP-SST cell density and molecular layer fiber density were found (data not shown). Within each group, the area occupied by eGFP+ processes was greater in the outer molecular layer compared to both the inner and middle lamina (Tukey’s multiple comparisons test; Control: Inner vs. Outer, *p* = 0.0011, Middle vs. Outer, *p* = 0.0428; DGC-Pten KO: Inner vs. Outer, *p* < 0.0001, Middle vs. Outer, *p* = 0.0049). This is consistent with the anatomy of HIPP cells, which innervate granule cell dendrites in the outer lamina (Milner and Bacon, 1989; Leranth et al., 1990). Finally, the lack of an effect of genotype on eGFP+ fiber density is notable because the area of the molecular layer was significantly increased in DGC-Pten KO mice relative to controls (Figure 7C; Control, 2.29e5 ± 9.40e3 μm^2^; KO, 2.90e5 ± 1.08e4 μm^2^; *p* = 0.0024), consistent with prior work in the model and likely reflecting hypertrophy of Pten KO granule cell dendrites (Arafa et al., 2019; Dusing et al., 2023). Preserved eGFP-SST fiber density in the face of tissue expansion and cell loss suggests that compensatory sprouting may be occurring.

### Immediate early gene expression among eGFP-SST interneurons and dentate granule cells

**FIGURE 8:**
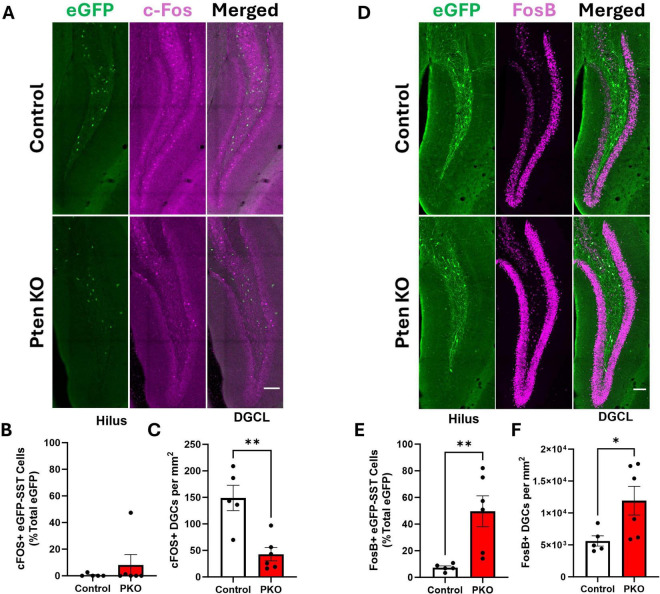
Altered Fos labeling of eGFP-SST interneurons and dentate granule cells in DGC-Pten KO mice. **(A)** c-Fos and eGFP-SST labeling in the dentate gyrus. Scale, 100 μm. **(B)** Percentage of hilar eGFP-SST interneurons with c-Fos immunostaining. **(C)** Density of c-Fos+ dentate granule cells. **(D)** FosB and eGFP-SST labeling in the dentate gyrus. Scale, 100 μm. **(E)** Percentage of hilar eGFP-SST interneurons immunoreactive for FosB. **(F)** Density of FosB+ dentate granule cells. *p < 0.05, **p < 0.01. Bars represent animal means ± SEM.

Anatomical findings suggest that surviving eGFP-SST neurons receive increased excitatory input from, and provide increased feedback inhibitory output to, dentate granule cells. We queried, therefore, whether eGFP-SST interneurons and DGCs in DGC-Pten KO mice would exhibit altered activity patterns. As an indirect measure of cellular activity, we immunostained for the immediate early gene c-Fos. c-Fos is rapidly and transiently expressed with neuronal activity (Herrera and Robertson, 1996; Peng and Houser, 2005), providing useful “snapshots” of recent activity levels. Colocalization of c-Fos with eGFP-SST cells was rare among both cohorts (Figures 8A, B), and did not differ significantly between groups (Mann–Whitney *U* = 13.0, *n*_1_ = 5, *n*_2_ = 6, *p* = 0.8485). c-Fos labeling of dentate granule cells, however, was significantly reduced in DGC-Pten KO mice relative to controls (Figure 8C; Control, 149 ± 24 cells/mm^3^; KO, 43 ± 13 cells/mm^2^; *p* = 0.0025). Findings suggest that at the time of sacrifice, Pten KO hippocampi were less active than normal.

As an additional indirect measure of activity, we immunostained for FosB. The FosB splice variant, ΔFosB, integrates activity over a longer temporal window due to its longer half-life (Chen et al., 1997). The proportion of FosB+, eGFP+ cells was significantly increased in DGC-Pten KO mice (Figures 8D, E; Control, 7.36 ± 1.24%; KO, 49.63% ± 11.54%; *p* = 0.0092). The proportion of FosB+ DGCs in DGC-Pten KO mice was also increased (Figure 8F; Control, 29.19% ± 4.13%; KO, 66.74 ± 10.42; *p* = 0.0127). The seemingly paradoxical c-Fos and FosB results likely reflect the different protein half-lives. The short half-life of c-Fos captures recent activity, likely reflecting longer interictal periods when activity levels drop below baseline. This pattern has been seen in other epilepsy models (Peng and Houser, 2005). With a half-life of days, on the other hand, ΔFosB likely reflects peak activity driven by daily seizure events. Taken together, c-Fos and FosB findings suggest that SST interneurons and DGCs have low activity during interictal periods but are recruited by seizure events or epileptiform activity.

## Discussion

Loss of inhibitory interneurons is common in epilepsy, with SST interneurons being especially vulnerable. Studies of interneuron loss, however, have largely relied on status epilepticus (SE) and traumatic brain injury (TBI) models, in which interneuron death occurs quickly after the insult. Here, we utilized a genetic mTORopathy model and modified it to enable fate-mapping of SST interneurons. In this model, loss of hippocampal SST neurons occurs secondary to Pten loss from excitatory granule cells, while surviving SST neurons show evidence of increased size, innervation, and activity. Delayed loss of interneurons may better recapitulate the pathogenesis in many patients with epilepsy, who often have no history of SE or brain injury. Elucidating the mechanisms leading to interneuron loss and sprouting in this model may provide novel therapeutic insights for epilepsy.

### SST interneuron loss in the DGC-Pten KO model

Interneuron loss is a hallmark pathology of epilepsy, evident in almost all animal models of the disease and across a broad diversity of epilepsy types in patients. This loss is hypothesized to play a causal role in many epilepsies by disrupting excitatory/inhibitory balance in the brain. Questions remain, however, about how this interneuron loss occurs. The mechanisms of interneuron loss have been predominantly examined in status epilepticus models, in which neurons die within days of this acute brain insult. In humans, however, epilepsy often develops in the absence of an acute neurological event, such as SE or TBI. Developing more realistic models that reproduce interneuron loss, therefore, is essential to understanding this epileptogenic event.

Here, we deleted the mTOR pathway inhibitor Pten from a subset of excitatory hippocampal granule cells, modeling the mosaic loss of mTOR regulators now identified in focal cortical dysplasia Type II (FCDII), tuberous sclerosis complex, and numerous other conditions associated with epilepsy (Girodengo et al., 2022). Somatic mutations in neural progenitor cells appear to underlie the focal abnormalities in these mTORopathies (Martin et al., 2017; Baldassari et al., 2019), and evidence for a similar mechanism of somatic Ras pathway mutations has been identified for idiopathic temporal lobe epilepsy (Khoshkhoo et al., 2023). Notably, while mutations are hypothesized to be restricted to excitatory cells, pathological specimens consistently show interneuron loss in FCD (Spreafico et al., 2000; Zamecnik et al., 2006; André et al., 2010). We now present strong evidence for a similar phenomenon in the DGC-Pten KO model. Prior stereological studies revealed a reduction in the number of hilar SST (HIPP) neurons in DGC-Pten KOs (LaSarge et al., 2021), which has now been confirmed using the eGFP fate-mapping approach. Importantly, this new approach is not dependent on SST protein levels, which can be downregulated, such that cells might still be present but undetectable with immunohistochemistry.

An unresolved issue raised by the present study is establishing when the SST neurons die. Control and DGC-Pten KO mice are functionally identical until P14, when tamoxifen treatment is given to activate cre recombinase and delete Pten. Generation of SST interneurons occurs embryonically, so both groups should start with identical cohorts of interneurons (Rapp and Amaral, 1988). SST loss, therefore, presumably occurs over the protracted period between 2 and 10 weeks of age, when tissue was collected. Animals do differ by the presence or absence of cre (Gli1-CreER^T 2+/–^), but prior work with cre+ controls showed no effect of cre alone on SST interneuron numbers (LaSarge et al., 2021). At 10 weeks, remaining eGFP-SST cells appeared healthy, implying that loss occurred earlier and dead cells had been cleared. Protracted non-convulsive hippocampal seizures have been observed in the DGC-Pten KO model (Pun et al., 2012), and it is possible that these focal seizures produced an abrupt loss of SST interneurons. Alternatively, loss could occur at such a slow rate that the odds of “catching” a cell at the time of death are low. Notably, even if cell loss is delayed until after seizure onset at around 6 weeks (Pun et al., 2012), that still leaves a month during which cell loss might occur. Microglial clearance of dead neurons can be completed in days (Balena et al., 2023), so loss of a large cohort of SST neurons at an earlier timepoint, or gradual dropout over weeks could make it difficult to pinpoint the exact time with cell death markers. Nonetheless, establishing the rate of cell loss in the DGC-Pten KO model would provide important insights for how similar interneuron loss might occur in patients with epilepsy.

A further unresolved issue is establishing why SST interneurons die. Loss of eGFP-SST HIPP cells in the dentate hilus is perhaps expected, as these neurons are directly innervated by Pten KO DGCs, and might succumb to excitotoxic injury or localized inflammation, for example. More surprising is the loss of CA1 eGFP-SST neurons [mostly oriens-lacunosum moleculare (O-LM cells)], and preservation of interposed eGFP-SST cells in CA3. This makes it unlikely that all SST interneuron loss is occurring through direct effects of mTOR hyperactive DGCs. Why some downstream SST interneuron populations are lost while others are spared is not clear. Seizure-induced cell loss remains an obvious suspect, however, in contrast to SE models, the DGC-Pten KO model does not result in SE and convulsive seizures are relatively infrequent (although focal hippocampal seizures could be more common). While it is well established that SE damages interneurons, the extent to which single or infrequent seizures cause cell death remains uncertain (Henshall and Meldrum, 2012). Determining whether SST interneurons succumb to repeated seizures or other mechanisms, therefore, will require further studies. Notably, clinical relevance is supported by observations of progressive brain volume loss in patients with uncontrolled epilepsy (Whelan et al., 2018; Woźniak et al., 2022), making the DGC-Pten KO model useful for studying mechanisms of neuron loss.

### SST interneuron sprouting in the DGC-Pten KO model

Hippocampal granule cells receive their primary excitatory inputs from neurons in entorhinal cortex via the perforant path. In the healthy brain, granule cells fire infrequently, and are further controlled by robust feedforward and feedback inhibitory circuits. SST neurons in the hilus (HIPP cells) are a critical component of this inhibitory circuitry. The present study reveals a substantially modified circuit in DGC-Pten KO mice. Granule cell input to hilar interneurons, revealed by Znt3 labeling of their presynaptic terminals, appears to be increased, while SST output to granule cells, based on axon density measurements, appears to be preserved despite loss of some SST cells and increases in molecular layer volume. Furthermore, SST interneuron activity, assessed by FosB immunostaining, may be increased. Despite these changes, which are predicted to preserve inhibitory tone in the dentate, prior electrophysiology studies in DGC-Pten KO model show that slices exhibit circuit level hyperactivity (LaSarge et al., 2021), KO cells receive increased excitatory and reduced inhibitory spontaneous postsynaptic currents (Santos et al., 2017), and animals have spontaneous seizures (Pun et al., 2012). It is possible that compensatory changes among SST interneurons are insufficient to offset hyperactive Pten KO cells. Alternatively, SST-mediated inhibition could be effectively recovered, and the decreased inhibition instead reflects changes occurring in other interneuron populations. Another possibility is that rather than being compensatory, the changes observed among eGFP-SST interneurons could be pathological and contribute to epileptogenesis, as has been suggested by a few studies implicating inhibitory interneurons in seizure initiation (Magloire et al., 2019; Wengert et al., 2021). Anatomical approaches make clear predictions about how circuits are altered, but future electrophysiological studies are needed to discriminate among these possibilities. An additional caveat is that the anatomical approaches used here do not distinguish between inputs from wildtype or Pten KO granule cells. Pten loss is associated with changes in synaptic properties (Weston et al., 2012; Barrows et al., 2017), so the source of Znt3+ inputs to SST interneurons remains to be established, as well as whether input to and from Pten KO cells functions normally.

## Conclusion

This study utilizes a model relevant to both temporal lobe epilepsy and mTORopathies in which deletion of Pten from a subset of dentate granule cells precipitates epilepsy development. We provide evidence of SST interneuron loss in multiple hippocampal regions, including the first assessments of interneuron density in CA1 and CA3 using this model. Interneuron loss occurring secondary to the primary insult (DGC Pten deletion) may reflect a more typical sequence of pathogenesis in patients with epilepsy. SST interneurons exhibited somatic hypertrophy, while immunohistochemical markers suggested increased activity and innervation. Similar changes among SST interneurons have been identified in other epilepsy models with disparate etiologies (Sloviter, 1987; Buckmaster and Dudek, 1997; Zhang et al., 2009), suggesting that this population of interneurons may be particularly important for maintaining excitatory/inhibitory balance in the face of insult.

## Data Availability

The raw data supporting the conclusions of this article will be made available by the authors, without undue reservation.
